# Indium gallium zinc oxide channel-integrated crystallization for wake-up-free HfZrO_x_ metal–ferroelectric–semiconductor gate stacks

**DOI:** 10.1080/14686996.2026.2740253

**Published:** 2026-10-12

**Authors:** Min-Kyu Kim, Ik-Jyae Kim, Jang-Sik Lee

**Affiliations:** Department of Materials Science and Engineering, Pohang University of Science and Technology (POSTECH), Pohang, Republic of Korea

**Keywords:** Hafnia ferroelectrics, indium gallium zinc oxide, channel-integrated crystallization, wake-up-free operation, partial polarization switching, ferroelectric thin-film transistor

## Abstract

Multilevel programming in ferroelectric neuromorphic hardware benefits from a well-defined initial polarization state; wake-up-induced polarization evolution can cause the same programming pulse to produce different polarization states during early operation. Here, we identify the semiconductor/ferroelectric integration sequence as a critical materials-design variable and demonstrate an indium gallium zinc oxide (IGZO) channel-integrated crystallization route for HfZrO_x_ metal–ferroelectric–semiconductor (MFS) gate stacks. The 10-nm-thick IGZO channel is deposited on 16-nm-thick HfZrO_x_ before annealing and retained during crystallization, enabling completion of the MFS gate stack with a single anneal and without sacrificial-metal removal. The completed stack exhibits a well-developed as-fabricated polarization–voltage (P–V) loop and only a 2.3% change in switchable polarization after 10^5^ bipolar cycles. By contrast, a complete sacrificial-TiN reference route shows a 39.0% wake-up increase followed by a 20.9% post-wake-up decrease. Starting from this well-defined as-fabricated polarization state, pulse amplitude, pulse width, and displacement-current compliance provide controlled access to intermediate polarization, including 16 current-controlled polarization levels. Ferroelectric thin-film transistors (FeTFTs) fabricated from the same gate stack convert the programmed polarization into four ordered threshold-voltage states. By linking the integration sequence to wake-up-free initialization, multilevel programming, and transistor-level readout, this work establishes a direct materials-to-function pathway relevant to oxide-semiconductor neuromorphic applications.

## IMPACT STATEMENT

IGZO channel-integrated crystallization enables single-anneal processing without sacrificial metal removal; comparison with a sacrificial TiN route links process sequence to wake-up-free as-fabricated polarization and multilevel FeTFT operation.

## GRAPHICAL ABSTRACT

**Figure:**
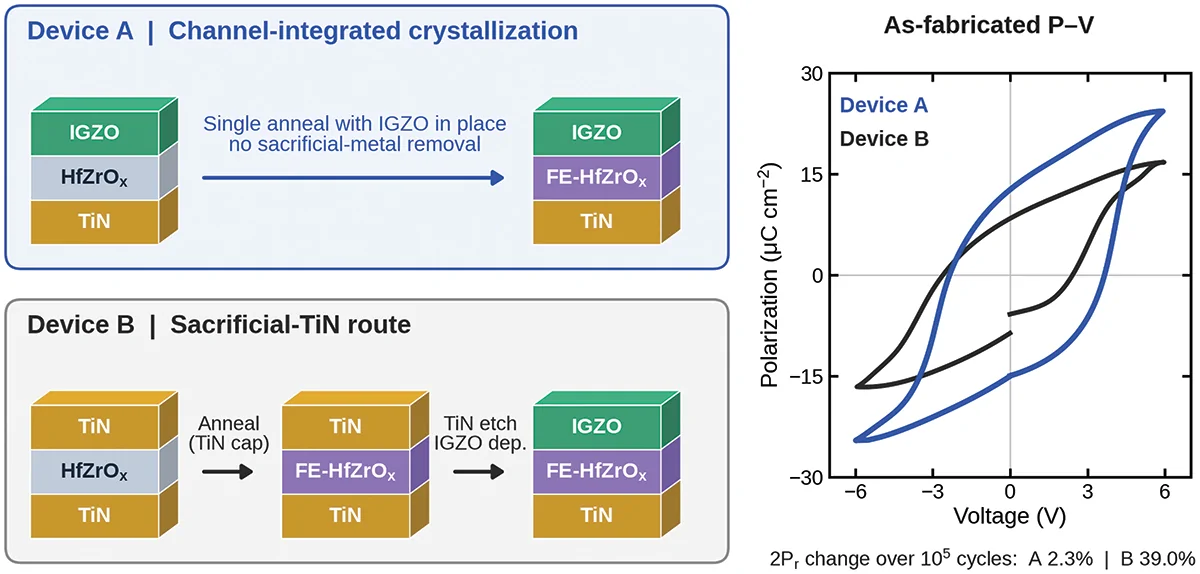

## 1. Introduction

Ferroelectricity in nanometer-scale HfO_2_-based films established fluorite-structured oxides as a scalable platform for nonvolatile electronics [1,2]. In HfZrO_x_, switchable polarization is associated primarily with a metastable non-centrosymmetric orthorhombic phase whose formation depends on composition, thickness, thermal processing, interfaces, and mechanical boundary conditions [3–5]. Atomic-layer-deposition compatibility, high coercive fields, and CMOS-compatible processing have consequently enabled HfO_2_-based capacitors and field-effect transistors for memory, in-memory computing, and neuromorphic hardware [6,7]. Three-terminal ferroelectric transistors are attractive neuromorphic devices because polarization states can be written through the gate and read through the semiconductor channel. Analog or multilevel weights are represented by partially switched domain configurations and can be accessed by controlling programming voltage, pulse width, or switching charge [8–12]. Such multilevel operation benefits from a well-defined initial polarization state. When switchable polarization evolves through wake-up, the same programming pulse can produce different states before and after wake-up cycling, increasing initialization and calibration overhead. Wake-up-free operation is therefore important for establishing a consistent pulse-to-state mapping.

Amorphous oxide semiconductors, particularly indium gallium zinc oxide (IGZO), are well suited to hafnia-based ferroelectric transistors because their electron density is efficiently modulated by polarization charge and their low-temperature deposition supports planar, back-end, and vertical integration [13–15]. Oxide-semiconductor-based ferroelectric transistors have demonstrated low-voltage memory and analog synaptic operation [11,12,16,17], while integrated arrays have extended the same materials platform toward compute-in-memory and three-dimensional neural-network hardware [18–20]. These developments place the semiconductor/ferroelectric interface at the center of both materials integration and weight-state readout. The as-fabricated switching state of HfZrO_x_ is established during crystallization. Capping-layer stiffness and thermomechanical constraint influence polar-phase stabilization [21–23], whereas oxygen-vacancy distributions, internal fields, and domain pinning govern whether the initial hysteresis loop is open or pinched [24–29]. Electrical cycling can redistribute charged defects and activate initially inactive switching regions, while continued cycling can promote interfacial charge accumulation, renewed pinning, and polarization loss [25–27,30]. Bulk-film defect engineering can suppress wake-up [28,29,31], and metal–ferroelectric–semiconductor (MFS) integration introduces an additional process variable through the sequence used to integrate the semiconductor with the ferroelectric layer.

Oxide-semiconductor/ferroelectric integration has been explored in IGZO-capped HfO_2_ capacitors, one-step-annealed ZrO_2_ transistors, oxide-assisted crystallization structures, and metal/IGZO-engineered HfZrO_x_ ferroelectric field-effect transistors (FeFETs) [20,32–35]. These studies demonstrate the feasibility of integrating oxide semiconductors with fluorite-structured ferroelectrics. Here, the integration sequence itself is treated as the experimental variable: the IGZO channel is deposited before HfZrO_x_ crystallization and retained in the completed MFS device, and this channel-integrated route is directly compared with a sacrificial-TiN integration route on the same HfZrO_x_ platform, in which IGZO is deposited after HfZrO_x_ crystallization. The channel-integrated route produces a well-developed as-fabricated hysteresis loop and maintains switchable polarization within 2.3% over 10^5^ bipolar cycles, whereas the sacrificial-TiN reference follows a pronounced wake-up-to-polarization-loss trajectory. Pulse-amplitude, pulse-width, and displacement-current-compliance measurements further establish controlled intermediate polarization, and ferroelectric thin-film transistors (FeTFTs) based on the same gate stack convert partial polarization into ordered threshold-voltage levels. This process-to-device sequence links control of the as-fabricated polarization state to multilevel operation relevant to neuromorphic applications.

## 2. Experimental

### 2.1. Fabrication of MFS capacitors and MFM references

MFS capacitors with an Al/IGZO/HfZrO_x_/TiN structure were fabricated on Si wafers with 100-nm-thick thermally grown SiO_2_. The substrates were sequentially sonicated in acetone, ethanol, and deionized water for 15 min each. A 100-nm-thick TiN bottom electrode was deposited by direct-current sputtering. A 16-nm-thick HfZrO_x_ film was deposited by atomic layer deposition at 280°C using alternating HfO_2_ and ZrO_2_ subcycles at a 1:1 cycle ratio. Tetrakis(ethylmethylamido)hafnium, tetrakis(ethylmethylamido)zirconium, and O_3_ were used as the Hf precursor, Zr precursor, and oxygen source, respectively.

For Device A, representing the channel-integrated crystallization route, a 10-nm-thick IGZO layer was deposited directly on the as-grown HfZrO_x_ film by radio-frequency (RF) sputtering at room temperature using an In:Ga:Zn = 1:1:1 target, an RF power of 150 W, and a working pressure of 5 mTorr. The IGZO/HfZrO_x_/TiN stack was annealed at 500°C for 1 min in N_2_, and the IGZO layer present during crystallization served as the semiconductor layer in the completed MFS device. For Device B, representing the sacrificial-TiN reference route, a 100-nm-thick TiN layer was deposited on HfZrO_x_ before annealing at 500°C for 1 min in N_2_. The TiN layer was removed using a 1:1 volume mixture of 30% NH_4_OH and 30% H_2_O_2_ at 50°C for 5 min. A 10-nm-thick IGZO layer was then deposited under the same sputtering conditions used for Device A, followed by a second anneal at 500°C for 1 min in N_2_. Finally, 100-nm-thick Al top electrodes with a diameter of 300 μm were deposited by electron-beam evaporation. Accordingly, the key distinction between the two routes is that the IGZO/HfZrO_x_ interface is present during HfZrO_x_ crystallization in Device A, whereas it is formed after TiN-assisted crystallization in Device B.

TiN/HfZrO_x_/TiN metal–ferroelectric–metal (MFM) capacitors were fabricated as reference structures using a 100-nm-thick TiN top electrode and the same HfZrO_x_ deposition and TiN-assisted crystallization conditions.

### 2.2. Fabrication of FeTFTs

Bottom-gate FeTFTs were fabricated using the Device A gate-stack sequence. A TiN gate electrode, a 16-nm-thick HfZrO_x_ layer, and a 10-nm-thick IGZO channel were formed sequentially, followed by annealing at 500°C for 1 min in N_2_. Al source and drain electrodes with a thickness of 100 nm were deposited by electron-beam evaporation. The channel length (*L*) and width (*W*) were 50 and 300 μm, respectively.

### 2.3. Structural and electrical characterization

The HfZrO_x_ and IGZO thicknesses were measured by spectroscopic ellipsometry (M-2000, J.A. Woollam, Co., Inc., USA) and atomic force microscopy (Dimension 3100, Veeco, Instruments Inc., USA), respectively. All electrical measurements were performed under ambient conditions at room temperature. Polarization–voltage (P–V) hysteresis, bipolar cycling, and pulse-dependent partial-switching measurements were carried out using a Keithley 4225-PMU (Keithley Instruments, USA). Device A and Device B were cycled using ±6 V pulses with a width of 4 μs. The switchable remanent polarization was defined as 2P_r_ = P_r_^+^ − P_r_^−^. The MFM reference was electrically woken up using 10^5^ bipolar pulses of ±5 V and 4 μs before post-wake-up cycling to 10^6^ cycles. Capacitance–voltage (C–V) measurements were performed at 100 kHz using an HP 4194A (Hewlett-Packard, USA) impedance analyzer.

For pulse-amplitude- and pulse-width-controlled switching, the MFS capacitor was initialized with a −6 V, 1 ms erase pulse. Positive programming pulses were applied with amplitudes from 2.1 to 6.0 V in 0.3 V steps and widths from 500 ns to approximately 146 μs in multiples of 1.5. The switched polarization, ΔP, was obtained from the difference between the charge collected during the first negative read pulse, which included switching and nonswitching components, and the charge collected during a second identical negative read pulse, which contained the nonswitching component. For displacement-current-compliance measurements, the capacitor was initialized using a −6 V, 10 ms pulse and programmed using a 6 V, 10 ms pulse while the displacement-current compliance was varied from 0.3 to 2.2 μA in 0.1 μA steps. Sixteen consecutive settings from 0.4 to 1.9 μA were used to evaluate multilevel polarization control.

FeTFT transfer characteristics were measured using a Keithley 4200A-SCS parameter analyzer (Keithley Instruments, USA). The gate voltage was swept between −4.5 and 4.0 V at drain voltages of 0.01, 0.05, and 0.1 V. The threshold voltage was extracted using a constant-current criterion of I_DS_ = 10^−7^ A × *W*/*L*. Multilevel operation was evaluated after a −4 V, 10 ms erase pulse using positive programming pulses of 2.5, 3.0, and 3.5 V with a width of 100 μs.

## 3. Results and discussion

### 3.1. Channel-integrated crystallization and the initial polarization state

Figure 1(a) compares the two MFS fabrication sequences. In Device A, IGZO is deposited before HfZrO_x_ crystallization and retained in the completed gate stack, which is formed with a single anneal. In Device B, HfZrO_x_ is crystallized beneath a sacrificial TiN layer; the TiN is then removed by wet etching, IGZO is deposited on the exposed HfZrO_x_ surface, and a second anneal is applied. Device A exhibits well-developed P–V hysteresis directly in the as-fabricated state (Figure 1(b)). The loop opens progressively with increasing sweep amplitude and reaches P_r_^+^ = 12.9 μC cm^−2^ and P_r_^−^ = −15.2 μC cm^−2^ at 6 V. No wake-up pulses were applied before this measurement. A direct comparison of the as-fabricated P–V responses at ±6 V is provided in Figure S1, showing the well-developed initial hysteresis of Device A and the pinched initial response of Device B. Device B was cycled for 10^5^ bipolar cycles before the voltage-dependent loops in Figure 1(c) were measured. After wake-up, P_r_^+^ and P_r_^−^ reach 10.1 and −9.7 μC cm^−2^, respectively, at 6 V. Thus, the two complete process sequences establish markedly different initial switching states despite using the same HfZrO_x_ deposition and primary annealing temperature.

**Figure 1.**
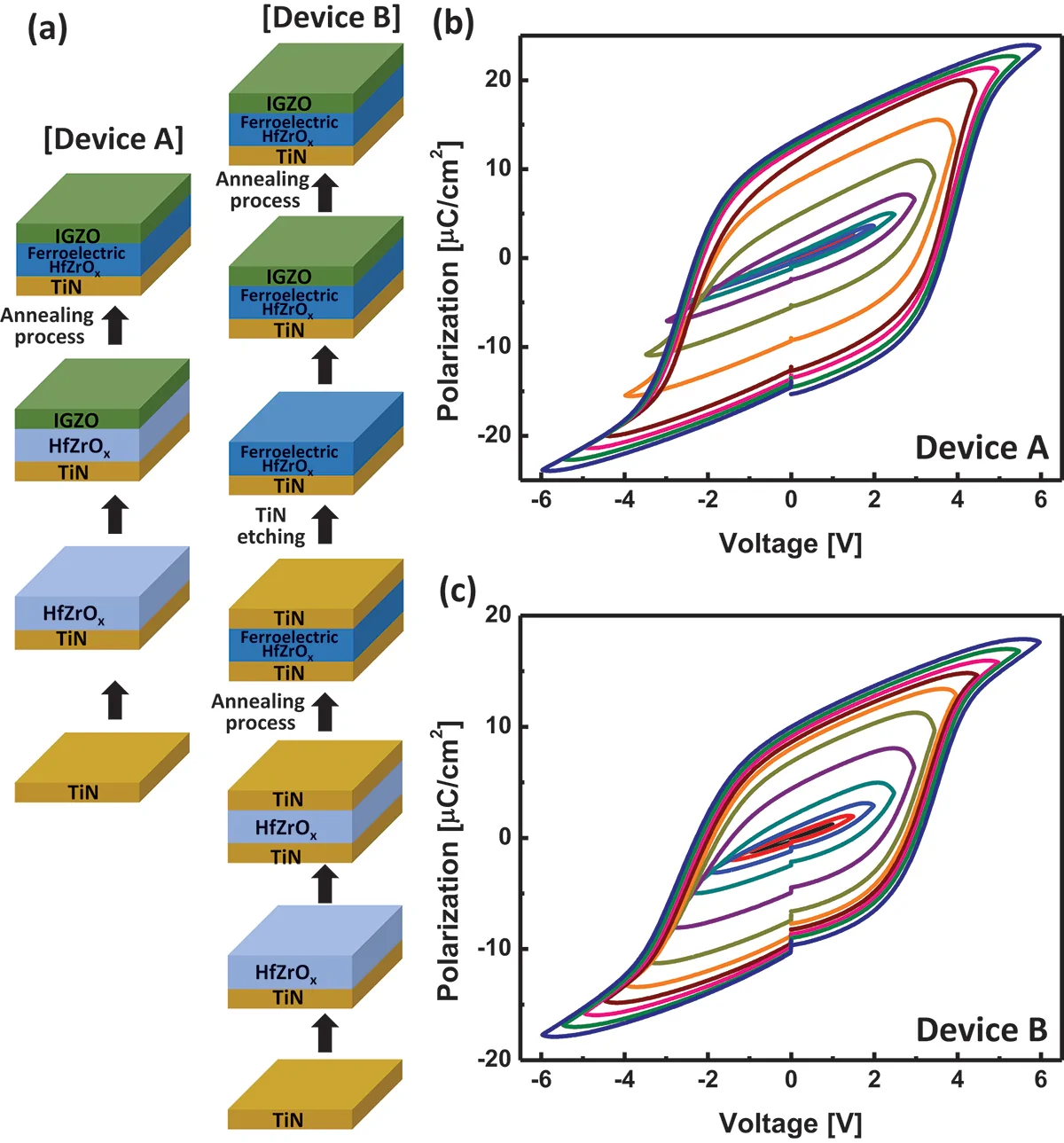
Fabrication routes and polarization characteristics of the MFS capacitors. (a) Process sequences of Device A and Device B. (b) As-fabricated P–V loops of Device A. (c) P–V loops of Device B after 10^5^ wake-up cycles.

The two integration routes establish different boundary conditions and interface histories. Mechanical confinement and capping-layer elasticity modify the stress state relevant to polar-phase stabilization [21–23], while adjacent-layer chemistry influences oxygen exchange, charged-defect distributions, and local internal fields [24–27]. In Device A, IGZO is present during HfZrO_x_ crystallization and remains in the completed device. In Device B, HfZrO_x_ is crystallized against TiN, followed by TiN removal, IGZO deposition, and a second anneal. These differences provide the process context for the distinct as-fabricated polarization responses of Devices A and B.

### 3.2. Wake-up-free operation and the cycling trajectory of the integration routes

Figure 2 compares the evolution of polarization for the two MFS integration routes. Device A maintains nearly constant P_r_^+^ and P_r_^−^ values throughout 10^5^ cycles under ±6 V, 4 μs pulses (Figure 2(a)). Its as-fabricated and post-cycling P–V loops are nearly superimposed (Figure 2(b)), and 2P_r_(10^5^)/2P_r_(0) = 1.023. Wake-up-free operation is defined here by the well-developed ferroelectric response before electrical cycling; the subsequent 2.3% change shows that this as-fabricated state also provides a stable early-cycle baseline. Device B follows a two-stage trajectory. During the first 10^5^ cycles, the pinched loop opens and 2P_r_ increases by 39.0%, corresponding to 2P_r_(10^5^)/2P_r_(0) = 1.390 (Figure 2(c)). During the following 10^5^ cycles, 2P_r_ decreases to 79.1% of the post-wake-up value, giving 2P_r_(2 × 10^5^)/2P_r_(10^5^) = 0.791 (Figure 2(d)). Device B therefore shows a substantial wake-up stage followed by a polarization-loss regime. Device A begins close to its measured early-cycle operating state. The TiN/HfZrO_x_/TiN MFM reference in Figure S2 also undergoes TiN-assisted crystallization and exhibits wake-up, followed by little change in remanent polarization during subsequent cycling to 10^6^ cycles. This contrast indicates that TiN-assisted crystallization alone does not account for the post-wake-up polarization decrease observed in Device B; the behavior should therefore be considered in the context of the complete sacrificial-TiN MFS integration sequence.

**Figure 2.**
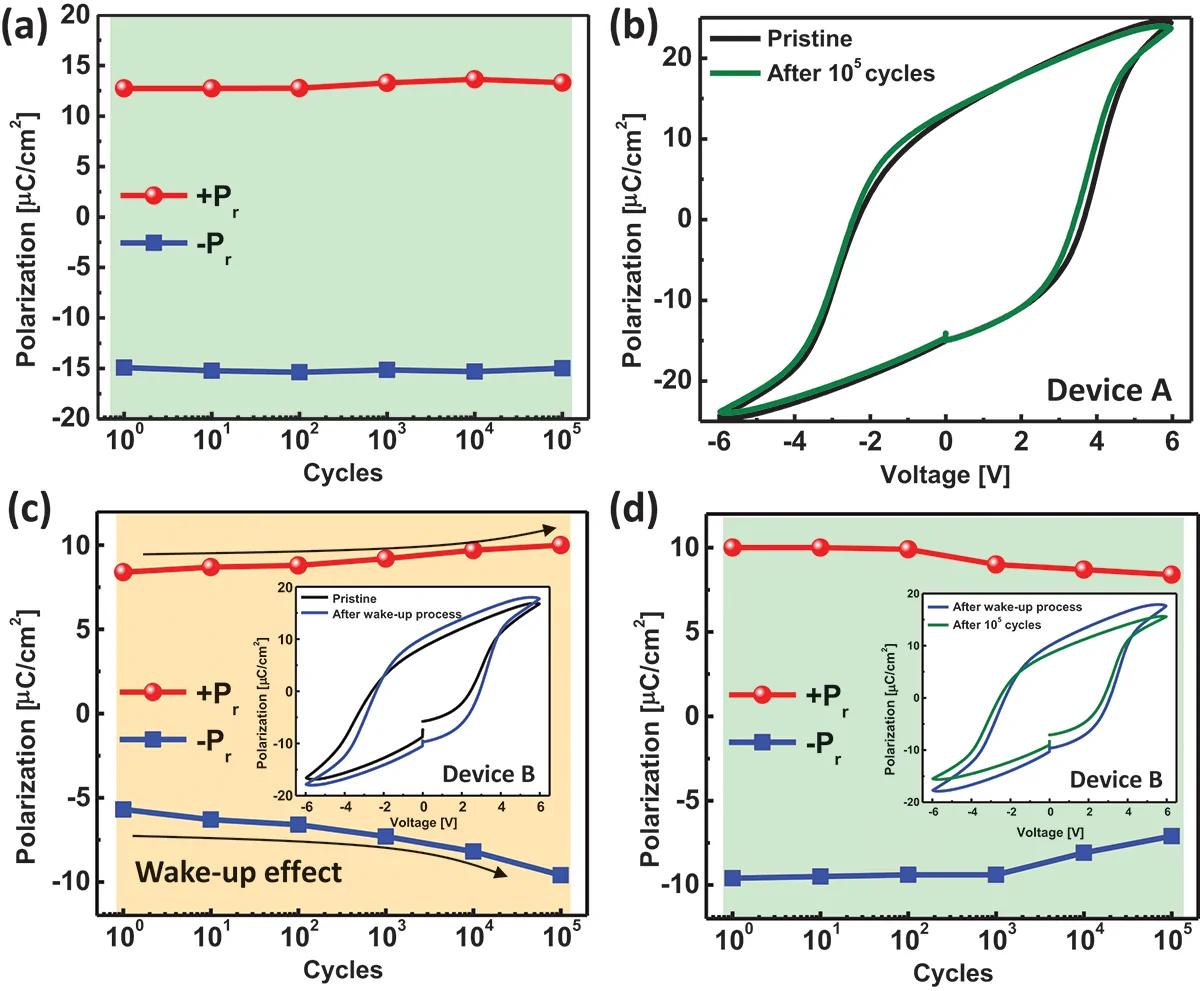
Wake-up and cycling characteristics of the MFS capacitors. (a,b) Polarization of Device A during 10^5^ cycles. (c) Wake-up of Device B. (d) Post-wake-up polarization decrease of Device B.

The pinched as-fabricated loop of Device B and its 39.0% increase in switchable polarization during the first 10^5^ cycles are consistent with the wake-up behavior widely reported for HfZrO_x_ [24–29]. During continued cycling, Device B subsequently shows a decrease in switchable polarization, consistent with the polarization-loss behavior reported for hafnia ferroelectrics [25–27,30].

For multilevel programming, the practical consequence of suppressing wake-up is the stability of the starting polarization state. Device B changes its switchable polarization by 39.0% during the initial wake-up stage, whereas Device A changes by only 2.3% over the same cycle range. Device A therefore provides a well-defined initial reference for pulse-controlled polarization updates. This stable starting condition supports a more consistent pulse-to-state mapping and is directly relevant to multilevel programming in neuromorphic applications.

### 3.3. Partial-polarization control for multilevel programming

The channel-integrated MFS stack was next evaluated for controlled access to intermediate polarization states. Figure 3(a) shows ΔP as a function of programming amplitude for pulse widths from 500 ns to approximately 146 μs, while Figure 3(b) shows the response as a function of pulse width at selected amplitudes. At a fixed pulse width, increasing the amplitude increases the switched fraction; at a fixed amplitude, increasing the duration produces the same monotonic trend. The voltage–time programming space therefore supports both rapid large updates at high field and finer updates at lower amplitude or shorter duration. Displacement-current compliance provides a third programming axis. Increasing the current limit progressively opens the P–V loop (Figure 3(c)), because the current limit constrains the charge delivered during polarization reversal. Across 16 consecutive current settings from 0.4 to 1.9 μA, ΔP increases monotonically and approximately linearly (Figure 3(d)), demonstrating controlled access to 16 current-controlled polarization levels. This result directly demonstrates control of the switched fraction in the charge domain. Voltage-, time-, and charge-controlled programming address complementary aspects of multilevel polarization updates. Voltage selects the driving field, pulse width determines the available switching time, and current compliance constrains the polarization increment during the switching event. Deterministic polarization control and engineered FeFET gate stacks have been used to improve multilevel separation and weight precision [9,36–38]. Here, all three programming modes are referenced to the same wake-up-free as-fabricated state, linking the process-defined initial condition to the programmed polarization response.

**Figure 3.**
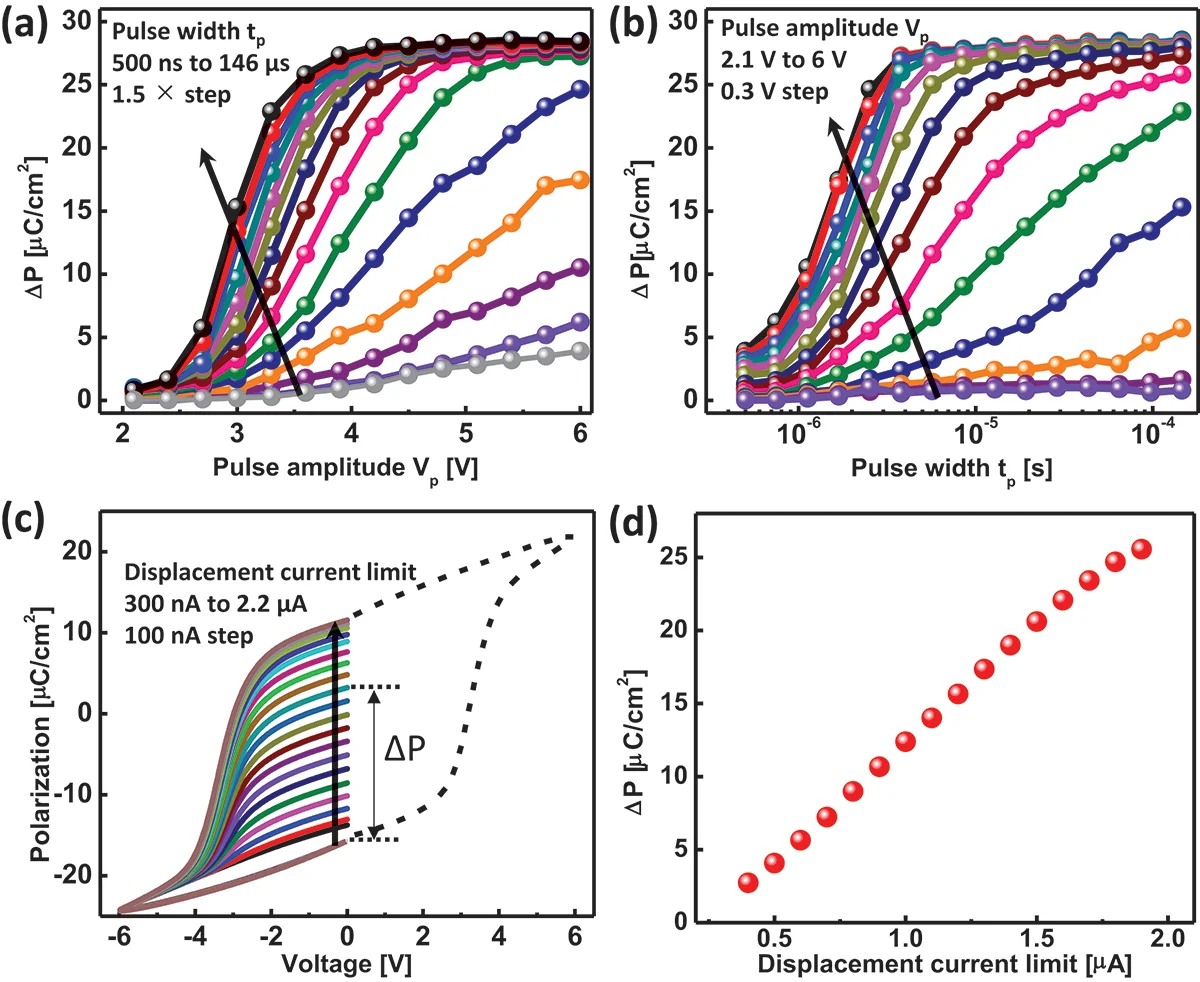
Partial-polarization control in Device A. (a,b) Pulse-amplitude- and pulse-width-dependent partial switching. (c) P–V loops under displacement-current compliance. (d) Sixteen current-controlled polarization levels.

### 3.4. Polarization-to-channel coupling and multilevel FeTFT readout

The C–V characteristics provide an electrical link between ferroelectric switching and semiconductor response (Figure S3). The TiN/HfZrO_x_/TiN MFM capacitor exhibits the expected butterfly-like hysteresis associated with polarization reversal. The IGZO/HfZrO_x_/TiN MFS capacitor exhibits a modified hysteretic response because the measured capacitance includes both the ferroelectric contribution and voltage-dependent accumulation or depletion in the n-type IGZO layer. The MFS response shows that the IGZO layer retained through crystallization remains electrostatically coupled to HfZrO_x_ polarization.

The same channel-integrated gate stack was implemented in bottom-gate FeTFTs (Figure 4(a)). Transfer characteristics measured at V_DS_ = 0.01, 0.05, and 0.1 V exhibit anticlockwise hysteresis (Figure 4(b)), consistent with polarization-controlled electron accumulation and depletion in the IGZO channel. The clear gate modulation after the 500°C crystallization anneal confirms that the IGZO layer remains a functional semiconductor while serving as the layer present at the HfZrO_x_ surface during crystallization. The forward and reverse sweeps yield different threshold voltages, defining the memory window shown in Figure 4(c). Polarization toward the IGZO interface increases electron density and shifts V_th_ in the negative direction, whereas the opposite polarization shifts V_th_ positively. The resulting memory window converts the capacitor-level polarization state into an electrically readable transistor parameter. Its magnitude reflects the combined ferroelectric polarization, semiconductor capacitance, interfacial screening, and threshold-voltage electrostatics of the IGZO/HfZrO_x_ stack [6,7,16,20].

**Figure 4.**
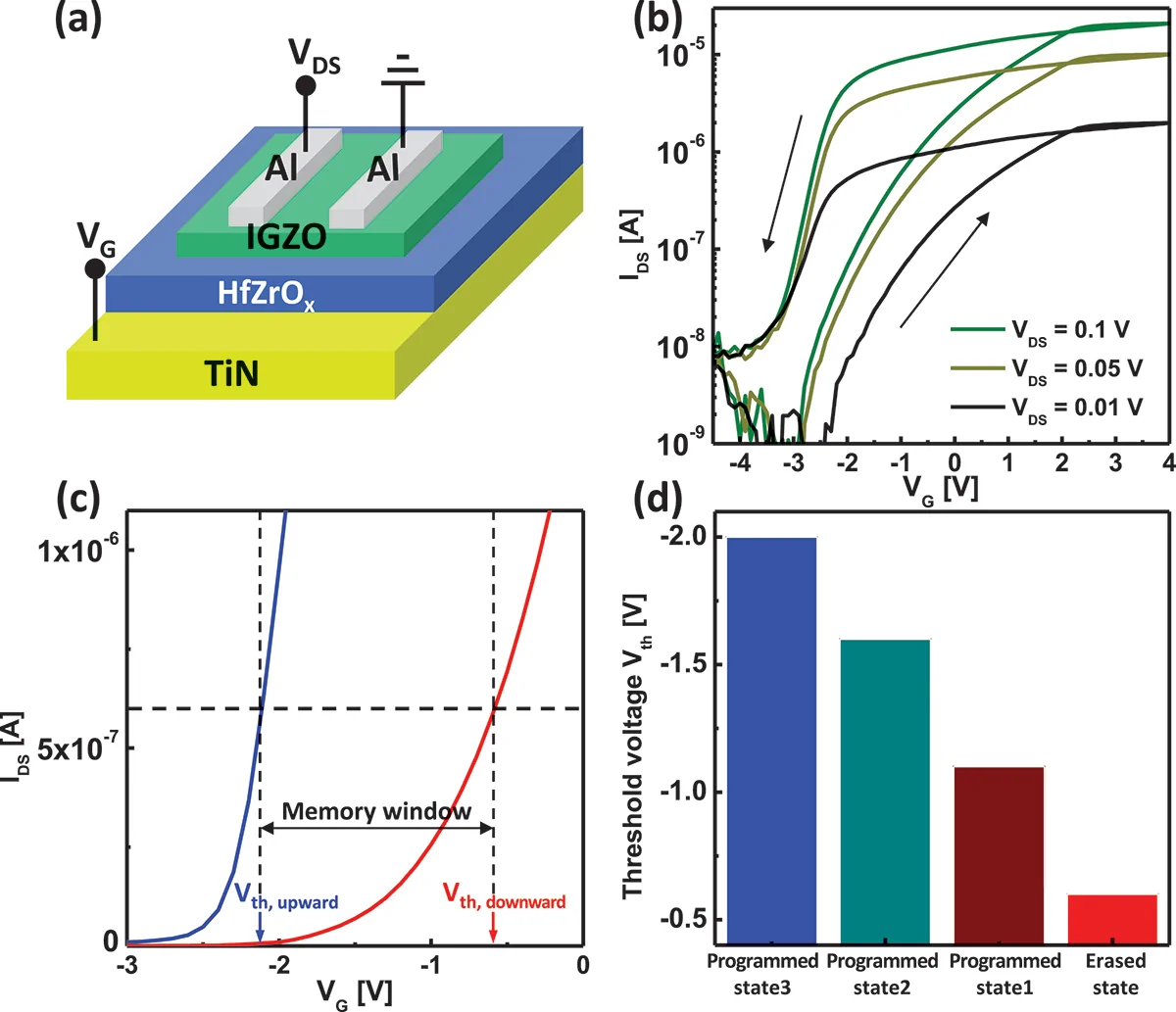
FeTFT readout of the channel-integrated gate stack. (a) Device structure. (b) Transfer characteristics at different drain voltages. (c) Threshold-voltage extraction and memory window. (d) Four ordered threshold-voltage states.

Partial-polarization programming generates four ordered V_th_ states (Figure 4(d)). A −4 V, 10 ms erase pulse establishes the reference state, and 2.5, 3.0, and 3.5 V program pulses with a common width of 100 μs produce monotonic negative shifts in threshold voltage. These transistor states extend the continuous capacitor-level polarization control of Figure 3 to electrically readable FeTFT operation. Together with the 16 current-controlled polarization levels, the result establishes a process-to-device pathway from a wake-up-free initial gate stack to multilevel FeTFT readout.

The complete integration sequence links three functions that are often optimized separately: crystallization of the ferroelectric layer, formation of the semiconductor interface, and conversion of partial polarization into readable transistor states. Channel-integrated crystallization therefore provides a direct route from process simplification to multilevel FeTFT operation using the as-fabricated gate stack.

## 4. Conclusions

An IGZO channel-integrated crystallization route was developed in which the oxide-semiconductor channel is deposited directly on HfZrO_x_ before annealing and retained in the completed MFS gate stack. Compared with the sacrificial-TiN route, the channel-integrated route produces a well-developed ferroelectric response directly in the as-fabricated state and changes its switchable polarization by only 2.3% during 10^5^ bipolar cycles. The sacrificial-TiN MFS device instead exhibits a 39.0% wake-up increase followed by a 20.9% decrease from its post-wake-up polarization. The channel-integrated gate stack further supports pulse-amplitude-, pulse-width-, and displacement-current-controlled partial switching, including 16 current-controlled polarization levels, and FeTFTs exhibit four ordered threshold-voltage states. These results establish the integration sequence as a practical design variable linking the as-fabricated polarization state to controlled multilevel FeTFT operation relevant to neuromorphic applications.

## Data Availability

The data that support the findings of this study are available from the corresponding author upon reasonable request.
